# Urology: a trip into metaverse

**DOI:** 10.1007/s00345-023-04560-3

**Published:** 2023-08-08

**Authors:** Gianmarco Randazzo, Giuseppe Reitano, Filippo Carletti, Massimo Iafrate, Giovanni Betto, Giacomo Novara, Fabrizio Dal Moro, Fabio Zattoni

**Affiliations:** Department Surgery, Oncology and Gastroenterology, Urologic Unit, University of Padova, 35122 Padua, Italy

**Keywords:** Metaverse, Urology, Virtual reality, Healthcare, Internet of things, 5G, Metaverse-assisted surgery

## Abstract

**Purpose:**

Metaverse is becoming an alternative world in which technology and virtual experiences are mixed with real life, and it holds the promise of changing our way of living. Healthcare is already changing thanks to Metaverse and its numerous applications. In particular, Urology and urologic patients can benefit in many ways from Metaverse.

**Methods:**

A non-systematic literature review identified recently published studies dealing with Metaverse. The database used for this review was PubMed, and the identified studies served as the base for a narrative analysis of the literature that explored the use of Metaverse in Urology.

**Results:**

Virtual consultations can enhance access to care and reduce distance and costs, and pain management and rehabilitation can find an incredible support in virtual reality, reducing anxiety and stress and improving adherence to therapy. Metaverse has the biggest potential in urologic surgery, where it can revolutionize both surgery planning, with 3D modeling and virtual surgeries, and intraoperatively, with augmented reality and artificial intelligence. Med Schools can implement Metaverse in anatomy and surgery lectures, providing an immersive environment for learning, and residents can use this platform for learning in a safe space at their own pace. However, there are also potential challenges and ethical concerns associated with the use of the metaverse in healthcare.

**Conclusions:**

This paper provides an overview of the concept of the metaverse, its potential applications, challenges, and opportunities, and discusses the implications of its development in Urology.

## Introduction

Metaverse has been defined in many ways. Mostly all definitions fall into one of these four categories: Augmented Reality (AR), lifelogging, mirror world, and Virtual Reality (VR) [1].

Augmented reality involves enhancing the external world by incorporating additional layers of information and technology. This technology expands the real-world environment beyond an individual's physical surroundings using a location-aware system and interface [2].

Lifelogging involves augmenting the inner world by using smart devices to capture and record daily activities, which are then stored and accessible through the internet or smartphones. This practice has become increasingly popular, with platforms such as Twitter, Facebook, and Instagram serving as typical examples of lifelogging. Using of devices to record biometric information is lifelogging, too [1].

The mirror world is a virtual representation of the external world that involves an informationally enhanced simulation or "reflection" of reality. It can be thought of as a metaverse where the visual appearance and structural organization of the physical world are replicated in virtual reality, as if reflected in a mirror [2]. A representative example of the mirror world is video conferencing systems such as Zoom, Webex, Google Meet, and Teams [1].

Virtual reality is a component of the metaverse that creates a simulation of the inner world. This technology involves advanced 3D graphics and real-time communication tools; it enables users to experience a fully immersive virtual environment [1]. Another important part of virtual reality is the possibility for multiple users to access simultaneously and generate a 3D space in which everyone is represented by an avatar [3]. Recent advances in technology have made it possible to create fully realized metaverses that are accessible to a wide audience [4].

### Metaverse and healthcare

The potential for the Metaverse to revolutionize healthcare was demonstrated during the COVID-19 pandemic, when telemedicine consultations became widespread, allowing patients to receive care remotely [5]. However, the Metaverse offers even greater possibilities, for example, by enabling virtual consultations and remote real-time patient monitoring, surpassing the limits of telemedicine.

There is some more about Metaverse in healthcare. Virtual reality (VR) has already been used in pain management in various conditions, such as chronic diseases and recovery after surgery [6], and speaking about surgery, Metaverse could be a powerful ally in procedure planning and even during operations, thanks to 3D modeling [7] and Augmented Reality (AR) [2].

While the Metaverse has the potential to transform healthcare, it also holds promise in other fields such as education. Medical education and training, for example, could greatly benefit from Metaverse technology, particularly VR, which offers the key advantage of deep immersion and the possibility to learn in simulations, without putting actual patients at risk [8–11].

The emergence of 5G technology and the increasing interest in the Internet of Things (IoT) in healthcare are closely associated with the development of the Metaverse. With its vast range of potential applications, including remote real-time monitoring, platforms for chronic disease management, and rehabilitation, IoT holds immense promise for improving healthcare [12].

### Metaverse and urology

Urology has long relied on technology, particularly in the fields of robotic and endoscopic surgery. Beyond these applications, the Metaverse holds promise in other aspects of the field, including office and ward practice. Virtual reality and 3D modeling, for instance, offer tremendous potential for preoperative and intraoperative surgery. Furthermore, the Metaverse has the power to transform the way Urology and surgical procedures are learned, as well as how urological patients interact with specialists, such as through virtual consultations.

Our trip into Metaverse will begin by examining its potential benefits for patients. Specifically, we will explore virtual consultations and virtual offices, pain management, and rehabilitation. We will then shift our focus to urologists, investigating how the Metaverse can transform surgery planning, training, and education, particularly for young professionals and residents, and how Metaverse can help in research, for example, in virtual clinical trials. Metaverse can help keep urologists in touch one another, but it can give something more with virtual meetings. Finally, we will discuss about ethical considerations, as is important for healthcare professionals to consider the potential benefits and risks of this technology in Urology.

## Methods

A non-systematic literature review identified recently published studies dealing with Metaverse. The database used for this review was PubMed, Google Scholar, and the identified studies served as the base for a narrative analysis of the literature that explored the use of Metaverse in Urology.

## From telemedicine to virtual consultations and virtual offices

Telemedicine and virtual consultations are becoming increasingly important in healthcare, especially after the COVID-19 pandemic [5, 16], in which traditional visits became a risk for the patients. Besides, telemedicine has potential regardless the risk of disease transmission during hospital access, because it can enhance adherence to follow-up and speed-up controls at a lower cost [17–19].

**Fig. 1:**
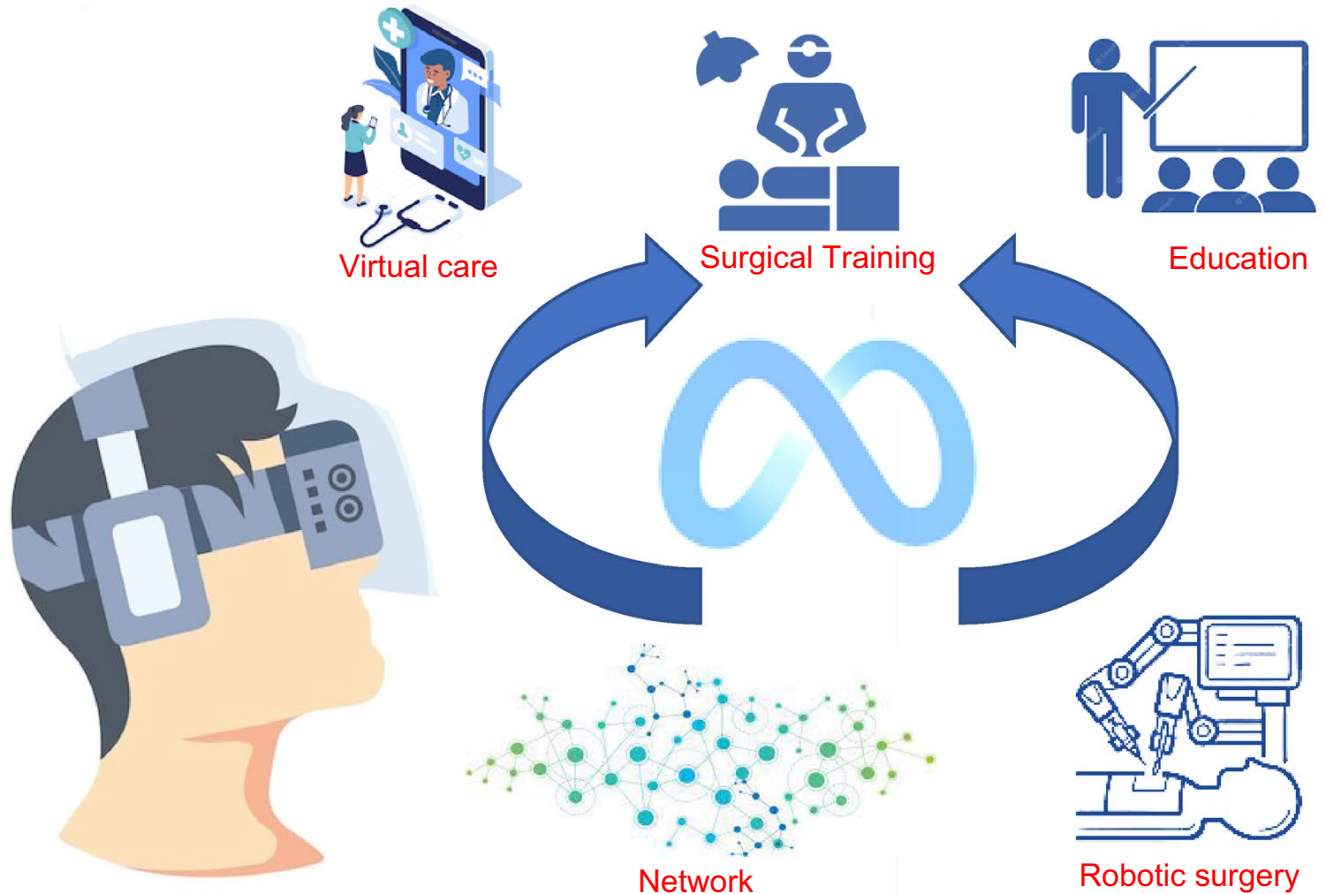
Main applications of metaverse in urology

In Urology, Metaverse can be used to create immersive virtual environments [13] where patients can interact with urologists in real time. This can be especially useful for patients who live in remote or rural areas and/or have difficulty accessing care. Urologists can enhance access to care, increase adherence to follow-up visits, and lower the risks associated with hospital access, such as the transmission of infections, by utilizing the Metaverse to revolutionize telemedicine and virtual consultations (Tables 1, 2, Fig. 1).

**Table 1:** Summary of articles dealing with metaverse, Urology, and healthcare, based on PubMed research of literature

References	Topic	Preclinical/clinical (n. of patients)	Description of application
Kye [1]	Metaverse definition	Preclinical	4 types of metaverse description
Smart [2]	Metaverse definition	Preclinical	Metaverse definition starting from web 2.0
Han [3]	Metaverse definition	Preclinical	Explore typologies of virtual world
Dwidedi [4]	Metaverse definition	Preclinical	Metaverse impact in real life
Bhugaonkar [13]	Metaverse definition	Preclinical	How Metaverse can promote innovative medical education, surgery, medical treatment, and online health management
Calvillo-Arbizu [12]	Metaverse definition	Preclinical	Explore application of IoT in healthcare
Gallagher [8]	Surgical training and education	Preclinical	Integration of VR training into a surgical training program
Nagendran [9]	Surgical training and education	Preclinical	Assess benefits and harms of supplementary virtual reality training of surgical trainees
Panait [10]	Surgical training and education	Preclinical	Developing of a laparoscopic skills curriculum based on a virtual reality simulator
Cevallos [11]	Surgical training and education	Preclinical	To examine the efficacy of VR to prepare surgical trainees for a pediatric orthopedic surgery procedure
Lungu [14]	Surgical training and education	Preclinical	Comprehensive overview of the application of VR, AR and MR for distinct surgical disciplines
Kim [15]	Surgical training and education	Preclinical	Investigate the state-of-the-art VR/AR technology relevant to plastic surgery
Novara [5]	Telehealth	Clinical (n. Pts = 34,350)	Telehealth in Urology: review of literature Telemedicine has been adopted successfully in selected patients with several common clinical urological conditions
Garfan [16]	Telehealth	Clinical (n/a)	Review of telehealth literature comprehensively since the pandemic started Need for establishing clear rules for telemedicine and regulations by governments and health organizations
Hatcher-Martin [17]	Telehealth	Clinical (n/a)	To determine potential cost-savings and patient acceptance of telemedicine A large number of patients want to participate in outpatient teleneurology visits
Russo [18]	Telehealth	Clinical (n. Pts = 5695)	Quantify money savings by adopting telemedicine in Veterans Affairs healthcare Telemedicine at the VA saves travel distance and time
Scott Kruse [19]	Telehealth	Clinical (n/a)	Evaluate barriers to adopting telemedicine worldwide through the analysis of published work Top barriers are technology specific and could be overcome through training and change management techniques
Alemayehu [20]	Telehealth	Preclinical	Opportunity with virtual or digital clinical trials in offering diverse patients easier and attractive means to participate in clinical trials
Inan [21]	Telehealth	Preclinical	Current state of the art for digital clinical trials
Skalidis [22]	Telehealth Education	Preclinical	Enhancing medical visits, assisting cardiovascular interventions, and reshaping the way medical education
Pandrangi [23]	Patient’s education	Clinical (n. Pts = 19)	Use of a 3D model of an abdominal aorta aneurysm to view in VR and so assess the use of VR in patient education VR proved to be an engaging learning tool that patients perceived as beneficial in understanding their health status
Gold [24]	Pain management	Clinical (n. Pts = 20)	Test the efficacy and suitability of virtual reality (VR) as a pain distraction for pediatric intravenous (i.v.) placement VR pain distraction was positively endorsed by all reporters and is a promising tool for decreasing pain, and anxiety in children undergoing acute medical interventions
Gold [25]	Pain management	Clinical (n/a)	Review of current literature on state-of-the-art pain distraction and future directions in VR VR pain distraction has become increasingly affordable, and initial research has demonstrated its efficacy for decreasing pain, anxiety, and fear
Furman [26]	Pain management	Clinical (n. Pts = 38)	Analgesic effect of immersive virtual reality (VR) during periodontal scaling and root planning (SRP) procedures Use of immersive VR distraction may be an effective method of pain control during SRP procedures
Schneider [27]	Pain management	Clinical (n. Pts = 137)	Predicting the difference between actual time elapsed during receipt of intravenous chemotherapy while immersed in a VR environment vs patient’s retrospective estimates of time elapsed VR is a non-invasive intervention that can make chemotherapy treatments more tolerable
Morris [28]	Pain management	Clinical (n/a)	Review evidence for the effectiveness of VR on reducing pain and anxiety in burn injury patients undergoing wound dressing changes and physiotherapy management VR could help manage burn pain and improve burn patient rehabilitation
Li [29]	Pain management	Preclinical	Exploring clinical and experimental applications of VR for acute and chronic pain management
Pourmand [6]	Pain management	Clinical (n. Pts = 460)	Virtual reality (VR) therapies as a clinical tool for acute and chronic pain Virtual reality can distract patients to reduce pain and anxiety
Vianez [30]	Rehabilitation	Clinical (n. Pts = 22)	Virtual reality exposure therapy (VRET) as an emerging treatment for people diagnosed with Post-Traumatic Stress Disorder (PTSD) Findings suggest VRET as a co-creation process, which requires more controlled, personalized, and in-depth research on its clinical applicability
Botelho [31]	Rehabilitation	Clinical (n. Pts = 46)	Pelvic floor muscle (PFM) training program designed as a virtual reality intervention program This virtual reality program promoted an increase in PFM contractility and a decrease in postmenopausal urinary symptoms
Carl [32]	Rehabilitation	Clinical (n. Pts = 1057)	Investigate efficacy of trials of virtual reality exposure therapy (VRET) for anxiety-related disorders VRET is an effective and equal medium for exposure therapy
Pires [7]	“Metaverse-assisted” surgery	Preclinical	Focus on VR as an alternative to slice-based medical analysis workstations
Profeta [33]	“Metaverse-assisted” surgery	Preclinical	Role of AR in relation to sentinel lymph node biopsy
Khor [34]	“Metaverse assisted” surgery	Preclinical	Introduction to the technology and the potential areas of development in the surgical arena of AR and VR
Jiang [35]	“Metaverse assisted” surgery	Preclinical	3D AR navigation method with point cloud-based image–patient registration to merge virtual images in the real environment for dental implants using a 3D image overlay
Vávra [36]	“Metaverse assisted” surgery	Preclinical	This review evaluates whether augmented reality can presently improve the results of surgical procedures
Pérez-Pachón [37]	“Metaverse assisted” surgery	Clinical (n/a)	Explore which existing tracking and registration methods and technologies allow healthcare professionals to develop and implement these systems in-house Need for more procedure-specific experiments with a sufficient number of subjects and measurements and including data about surgical outcomes and patients' recovery
Roberts [38]	“Metaverse assisted” surgery	Clinical (n/a)	Advance in the use of AR for improvements in urologic outcome Advances in AR have led to increasing registration accuracy as well as increased ability to identify anatomic landmarks and improve outcomes during urologic procedures such as RARP and robot-assisted partial nephrectomy
Onggirawan [39]	Surgical training and education	Preclinical	Using Metaverse in the form of virtual space in the educational field and how teachers and students respond to the process
Tan [40]	Virtual meetings	Preclinical	Creation of virtual environments with three-dimensional (3D) space and avatar
Kostick-Quenet [41]	Cybersecurity	Preclinical	Non-fungible tokens (NFTs) can help incentivize a more democratized, transparent, and efficient system for health information exchange in which patients participate in decisions about how and with whom their personal health information is shared
Jones [42]	Cybersecurity	Preclinical	NFTs definition and their use by scientists

*IoT* Internet of Things, *VR* Virtual Reality, *AR* Augmented Reality, *MR* Mixed Reality, *VA* Veterans Affairs, *PTSD* Post-Traumatic Stress Disorder, *PFM* Pelvic Floor Muscle, *NFTs* Non-Fungible Tokens, *RARP* Robot-Assisted Radical Prostatectomy, *VRET* Virtual Reality Exposure Therapy, *n. pts* number of patients included in this study

**Table 2:** Possible applications of metaverse in urology: pros and cons

Field of application	Pros	Cons
Virtual consultations	Reach rural areas, decrease time, and cost of visits	Elderly people not used to technology, cost of technology
Training and education	Learning anatomy in 3D, immersive surgical training, virtual congresses	Cannot replace real-life practice
Pain management	Deep immersion and reduction in opioid usage	Lack of evidence on long-term benefits
Surgical planning	3D anatomical modeling and procedure simulation	How well can models reproduce reality and intraoperative complications?
Rehabilitation	Improve adherence to exercise, reduce anxiety and promote well-being	Could generate dissociation from reality
Research	Virtual clinical trials	Not always possible (i.e., medical procedures or need for visit)
Meetings	Virtual meetings	Absence of human contact

### Virtual offices

There are various ways to create virtual offices, meeting the needs of both patients and doctors [13]. Depending on the patient's medical issue, different virtual offices can be created. For instance, if a patient has kidney stones, the office may feature informative material about the condition, including helpful advice and tips. Waiting rooms can be designed in a comparable manner, so that if patients have to wait for some time, they can be provided with an introduction to the relevant medical topic even before seeing the Urologist.

### Holograms

Patient–doctor communication is fundamental for the curative process [43], even though explaining to the patient his medical condition can be a challenge sometimes. However, in the Metaverse, holograms can be utilized to provide a more comprehensive explanation of anatomical issues and potential solutions. The use of 3D organ models and holographic demonstrations can be beneficial in educating patients and addressing their concerns [7]. The Metaverse can be a valuable tool for patient education, as it enables them to virtually explore their anatomy and gain a better understanding of their medical condition and upcoming surgical procedures [23]. By reducing anxiety and enhancing patient satisfaction, the Metaverse can help improve patient outcomes.

### Avatars

In cases of certain urological issues, patients may feel hesitant to describe their condition due to feelings of shyness or embarrassment (such as when discussing erectile dysfunction). Utilizing avatars may enhance communication between patients and doctors and facilitate the resolution of the most uncomfortable problems. Zepeto [44] is an excellent example of using avatars. It allows you to create your own avatar starting from a simple photograph. Thanks to artificial intelligence, your photo is processed, and you can then further personalize it [45].

Moreover, Metaverse can provide a platform for support groups and peer-to-peer counseling, allowing patients to connect with others who have experienced similar challenges and receive emotional support. The use of the Metaverse can also provide a space for patients to explore their treatment options and receive education about their condition, improving their overall health literacy and empowerment in decision-making.

## Pain management

The present outbreak of opioid abuse [46, 47] has intensified the need to find reliable pain relief methods that do not involve opioids [48]. Pourmand et al. [6] showed that virtual reality can be used as a distraction technique to reduce pain and anxiety. Their evidence indicates that using virtual reality is effective as short-term solution for pain relief in acute and chronic pain. In Urology, a useful application of this method could be seen in chronic pelvic pain syndrome and bladder pain syndrome.

Studies [24–28] have shown that VR can effectively reduce pain, anxiety, and unpleasantness associated with chemotherapy, lumbar puncture, port access, blood draw, and intravenous placement. VR distraction was significantly better than standard care in reducing physiological arousal and pain ratings and it can decrease symptoms distress and perceived time spent receiving chemotherapy with higher satisfaction levels [29].

As the cost of VR systems has decreased in recent years [49], immersive VR is becoming a more common adjunct therapy and may soon allow for patient-controlled pain relief in outpatient settings (Tables 1, 2).

## Rehabilitation

The success of rehabilitation is greatly influenced by emotional and psychological factors, and it is crucial for patients to feel comfortable and secure during therapy sessions. In this sense, Metaverse can provide a safe space, in which the patients can live immersive experiences that simulate real-world activities and environments, allowing them to practice activities of daily living and improve their functional abilities [13], practicing coping strategies and manage stress and anxiety [30] (Tables 1, 2).

Patients with urological conditions such as urinary incontinence and erectile dysfunction may experience psychological distress and anxiety related to their condition, which can impact their quality of life. Patients with urinary incontinence can use VR to practice pelvic floor muscle exercises [31, 50] and receive feedback on their performance, which can improve their bladder control and reduce their anxiety about incontinence. Similarly, patients with erectile dysfunction can use VR to practice relaxation techniques and mindfulness exercises to manage their anxiety and improve their sexual function [51]. Additionally, the Metaverse can provide a virtual environment for patients undergoing exposure therapy for sexual anxiety, allowing them to practice and build confidence in real-world scenarios [32].

## Surgical planning and beyond

Surgical planning is a critical step of any surgical intervention, as it allows surgeons to visualize the anatomy and plan the procedure. In traditional surgical planning, doctors rely on 2D images, mainly CT scans and MRIs. However, these images do not provide a complete view of the patient's anatomy, making it challenging to plan complex surgical procedures.

Immersive three-dimensional experiences offered by VR platforms have been demonstrated to assist in diagnosis. In a Metaverse platform, surgeons would generate a 3D virtual representation of the patient's anatomy, offering a more comprehensive view. The 3D model can be manipulated in real time, exploring different approaches and making more informed decisions [7].

Moreover, the Metaverse can serve as a platform for simulating the surgical procedure, enabling doctors to practice in a virtual environment before the actual operation (Fig. 1). This method can assist doctors in detecting possible complications and enhancing their surgical technique [14], ultimately resulting in improved outcomes for patients (Tables 1, 2).

The implementation of Metaverse technology can bring benefits to surgery, not only for preoperative planning but also for intraoperative use. Augmented reality has proven to be a valuable asset in utilizing 3D data during surgery [15, 33–37, 52], particularly in Urology, where it can aid in accurately identifying surgical margins for procedures such as robotic-assisted partial nephrectomy or robotic-assisted radical prostatectomy [38]. Additionally, it can assist in minimizing the risk of complications by indicating the location of fragile structures, making it a useful tool for both the procedure and safety measures [37]. These new tools can bring surgery to a new frontier, that we call “Metaverse-assisted surgery.”

## Education and training

The conventional approach in Medical School has been to study anatomy using atlases. While this method is steeped in tradition and the anatomical illustrations can be works of art, comprehending the intricacies of anatomy, particularly for intricate structures with a layered arrangement such as the heart, kidneys, and organ disposition in the abdomen, can be quite challenging. Moreover, it is often difficult for Medical Schools to organize cadaver sessions for both anatomy studying and surgery practice, due to availability and costs [13].

Metaverse can be used for immersive education and training experiences for medical students and professionals. Virtual reality has the advantage of immersing users in a learning environment that difficultly can be seen in reality, reaching a high level of active learning and personal presence [53].

By creating virtual surgeries, simulations, and interactive lectures, medical students and professionals can learn in a more engaging and interactive way. This can lead to better patient outcomes and improve the quality of care (Tables 1, 2).

### Residents and surgical training

An immersive learning context can make high-skilled surgeons even before touching a real patient. In fact, it is known that virtual reality simulation could improve surgical skills both in general and in specific in vivo situations [8–11]. Dangerous or high-risk activities, such as difficult surgeries can be reproduced through virtual simulation environments [39], improving learning experience. Residents can learn how to perform a particular procedure from zero, in a complete start to end simulation (Fig. 1). Artificial Intelligence (AI) could introduce each time some element of variation, as it happens in real life with real patients, and simulate as well intraoperative complications. Some companies have already started to work in this sense (i.e., Osso VR) [54].

## Research

With COVID-19 pandemic research had to face a new obstacle, leading to a slowdown in many areas, especially in clinical trials. FDA guidelines for the first time proposed alternative methods for collecting data (like phone contacts, virtual visits) [20, 55]. Additionally, prior to the pandemic, clinical trials were struggling with recruitment, data collection, and follow-up inefficiencies, resulting in increased costs and time [21]. Metaverse can be used for patient recruitment and retention in clinical trials, where patients can access and participate in virtual trials from anywhere in the world [20]. Virtual surveys, interviews, and patient feedback mechanisms are useful to collect this kind of data [21].

In Urology, a useful application of this method can be seen not only for clinical trials but also for collaborative research in multicenter studies, improving way of communication and sharing information and data (Tables 1, 2).

## Virtual meetings

Meetings and congresses are always great occasions to connect with other colleagues from other centers, but sometimes it can be difficult to be present, due to cost and time problems.

Metaverse can reduce the need for travel and increase accessibility to meetings and conferences for urologists and other healthcare professionals [40]. Virtual conferences can be held in a virtual auditorium or conference center, allowing attendees to listen to talks, participate in discussions, and ask questions (Fig. 1).

Moreover, the Metaverse can be used to host virtual exhibitions, which can be designed to provide a 3D representation of the products, allowing attendees to interact with them in a more immersive way [56]. Urologists can use the metaverse to host virtual workshops, allowing attendees to participate in hands-on training and learn new skills. Virtual environments can be designed to simulate a laboratory or surgical suite, allowing attendees to practice procedures and techniques in a virtual setting [1].

Finally, Metaverse can also be used for continuing medical education, where urologists can earn continuing education credits by participating in virtual lectures and simulations (Tables 1, 2)

### Ethics

As with any emerging technology, the use of the Metaverse in Urology raises ethical considerations. One important consideration is patient privacy and confidentiality. It is important to ensure that patient data are protected and secure, especially when using the Metaverse for telemedicine and virtual consultations. The informatics organization of the Metaverse working with blockchain could bring new ways of encrypting patient data and enforcing compliance to medical standards in practices and processes [22].

Non-fungible tokens (NFTs) could be a potential solution. NFTs are non-interchangeable unit of data, registered in a blockchain, that is used to record ownership of a digital asset [41, 42]. To enhance privacy, maintain data integrity, and preserve confidentiality in research endeavors, patient data such as medical histories, radiological, and laboratory examination results can be converted into NFTs through a process known as tokenization [22].

Another consideration is the potential for bias in the development and use of the Metaverse. It is important to ensure that the virtual environments and simulations accurately represent diverse patient populations and do not perpetuate harmful stereotypes or biases. Additionally, the use of the Metaverse in Urology raises questions about the appropriate use of technology in healthcare. It is important to consider the potential benefits and drawbacks of using the Metaverse for patient care, training, and research, and to ensure that the use of technology does not replace the human element of healthcare.

The integration of the Metaverse in the field of Urology poses concerns regarding the affordability and availability of healthcare services. Although the use of the Metaverse can enhance access to care for patients residing in remote or rural regions, it is vital to ensure that the cost of care remains reasonable and accessible for all patients.

In conclusion, it is crucial to contemplate the ethical implications of employing the Metaverse in the field of Urology and guarantee that its application aligns with the principles of medical ethics, such as respect for patient autonomy, beneficence, non-maleficence, and justice.

## Conclusions

The use of Metaverse in healthcare, and specifically in Urology, has enormous potential to revolutionize patient care, pain management, rehabilitation, educations and training, surgical practice, and research. With the ability to create immersive virtual environments, Metaverse can significantly enhance access to care, improve patient outcomes, and promote patients’ well-being in a way never explored before.

Virtual consultations allow patients to interact with urologists in real time, regardless of their location, and offer new ways to obtain detailed information about their condition, such as through hologram and 3D representation. With the help of virtual reality, the Metaverse has the potential to alleviate pain and anxiety related to urological conditions, such as bladder pain syndrome, and improve adherence to therapy, for example, by guiding patients through pelvic floor muscle training.

Immersive training and education experiences are another area where VR can be highly beneficial for urology students and professionals. The Metaverse offers a safe environment where they can practice at their own pace, which can enhance their learning experience. In the operating room, the Metaverse can be used both preoperatively, with 3D modeling and virtual simulations, and intraoperatively, with the assistance of AR and AI.

Virtual meetings and conferences can also be conducted in the Metaverse, making it a versatile and flexible tool for urology professionals. Additionally, even the research field can benefit from the Metaverse, thanks to virtual clinical trials.

However, as with any emerging technology, ethical considerations must be considered when using Metaverse in Urology. Therefore, it is important to approach its use thoughtfully, carefully consider the benefits and potential risks, and develop responsible policies and practices.

Overall, the Metaverse has the potential to transform the way urology professionals approach patient care, training, and surgery, and will undoubtedly play a significant role in shaping the future of the field.
